# Azido­{2-[bis­(2-hy­droxy­eth­yl)amino]­ethano­lato-κ^4^ *N*,*O*,*O*′,*O*′′}cobalt(II)

**DOI:** 10.1107/S1600536810047100

**Published:** 2010-11-20

**Authors:** Yan-Ju Liu, Huai-Xia Yang, Juan Yuan, Xia Wang

**Affiliations:** a Pharmacy College, Henan University of Traditional Chinese Medicine, Zhengzhou 450008, People’s Republic of China

## Abstract

In the title complex, [Co(C_6_H_14_NO_3_)(N_3_)] or [Co(teaH_2_)N_3_], the Co^II^ atom resides in a trigonal–bipymidal O_3_N_2_ environment formed by three O atoms and one N atom from a simply deprotonated tetra­dentate triethano­lamine ligand, and one N atom from an azide ligand. The O atoms define the equatorial plane whereas both N atoms are in axial positions. The mononuclear units are linked through O—H⋯O hydrogen-bonding inter­actions between the ethanol OH groups and the ethano­late O atom of a neighbouring complex into chains running parallel to [010].

## Related literature

For general background to complexes including teaH_3_ ligands, see: Liu, Wang *et al.* (2008 ▶); Liu, Zhang *et al.* (2008 ▶). For Co^II^ complexes with similar ligands, see: Malaestean *et al.* (2010 ▶).
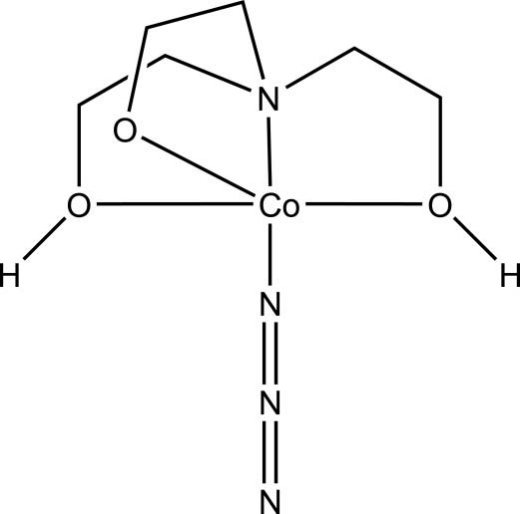


## Experimental

### 

#### Crystal data

[Co(C_6_H_14_NO_3_)(N_3_)]
*M* *_r_* = 249.14
Monoclinic, 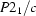

*a* = 8.7752 (2) Å
*b* = 7.9373 (1) Å
*c* = 14.4097 (3) Å
β = 107.084 (1)°
*V* = 959.37 (3) Å^3^
*Z* = 4
Mo *K*α radiation
μ = 1.78 mm^−1^
*T* = 293 K
0.20 × 0.20 × 0.10 mm

#### Data collection

Rigaku Saturn CCD diffractometer
Absorption correction: multi-scan (*REQAB*; Jacobson, 1998 ▶) *T*_min_ = 0.708, *T*_max_ = 0.823
4004 measured reflections
2179 independent reflections
1253 reflections with *I* > 2σ(*I*)
*R*_int_ = 0.055

#### Refinement

*R*[*F* ^2^ > 2σ(*F* ^2^)] = 0.038
*wR*(*F* ^2^) = 0.089
*S* = 0.89
2179 reflections
135 parameters
H atoms treated by a mixture of independent and constrained refinement
Δρ_max_ = 0.55 e Å^−3^
Δρ_min_ = −0.38 e Å^−3^

### 

Data collection: *CrystalClear* (Rigaku/MSC, 2006 ▶); cell refinement: *CrystalClear*; data reduction: *CrystalClear*; program(s) used to solve structure: *SHELXS97* (Sheldrick, 2008 ▶); program(s) used to refine structure: *SHELXL97* (Sheldrick, 2008 ▶); molecular graphics: *SHELXTL* (Sheldrick, 2008 ▶); software used to prepare material for publication: *publCIF* (Westrip, 2010 ▶).

## Supplementary Material

Crystal structure: contains datablocks I, global. DOI: 10.1107/S1600536810047100/wm2427sup1.cif

Structure factors: contains datablocks I. DOI: 10.1107/S1600536810047100/wm2427Isup2.hkl

Additional supplementary materials:  crystallographic information; 3D view; checkCIF report

## Figures and Tables

**Table 1:** Selected bond lengths (Å)

Co1—O3	1.991 (2)
Co1—N2	2.013 (3)
Co1—O1	2.064 (2)
Co1—O2	2.065 (2)
Co1—N1	2.148 (3)

**Table 2:** Hydrogen-bond geometry (Å, °)

*D*—H⋯*A*	*D*—H	H⋯*A*	*D*⋯*A*	*D*—H⋯*A*
O2—H1*OA*⋯O3^i^	0.80 (6)	1.80 (6)	2.595 (3)	176.90
O1—H2*OA*⋯O3^ii^	0.74 (3)	1.83 (3)	2.573 (3)	177.70

Symmetry codes: (i) 
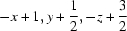
; (ii) 
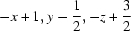
.

**Fig. 1.**
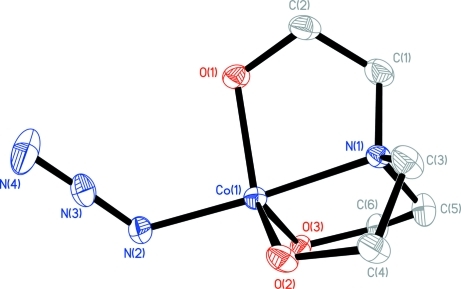
View of the molecular structure of (I), showing the labelling of the atoms drawn with displacement ellipsoids at the 30% probability level. All H atoms have been omitted for clarity.

**Fig. 2.**
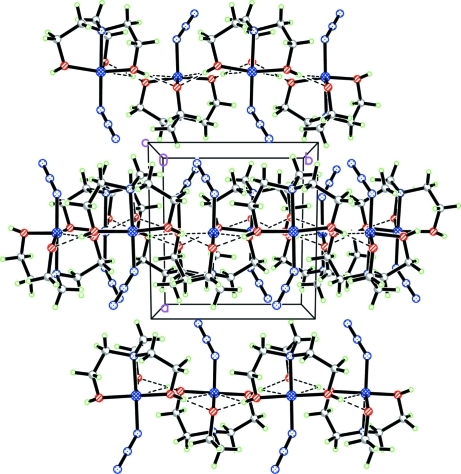
A view of the crystal packing along the c axis. Hydrogen bonds are indicated with dashed lines.

## supplementary crystallographic information

### Comment

The design and synthesis of mononuclear compounds with strong anisotropy, potentially acting as single ion magnets, are of current interest. Podand-like or multi-dentate ligands, such as diethanolamine (deaH_2_) or triethanolamine (teaH_3_), have been employed though these ligands were also used to prepare other kinds of clusters (Liu, Wang *et al.*, 2008; Liu, Zhang *et al.*, 2008). In this work, we selected teaH_3_ as a capping ligand, and azide as another anion, generating complex (I), Co(N(CH_2_CH_2_OH)_2_(CH_2_CH_2_O))N_3_ [= Co(teaH_2_)N_3_].

In the structure of (I) each Co^II^ atom is five-coordinate by three O atoms and one N atom from a simply deprotonated tetradentate triethanolamine ligand, and one N atom from an azide ligand in a trigonal-bipymidal coordination environment (Fig. 1). The O atoms define the equatorial plane whereas both N atoms sit in axial positions. The Co—N distances are 2.013 (3)—2.148 (3) Å, and the Co—O distances are 1.991 (2)–2.065 (2) Å. These bond length are in agreement with similar complexes with Co^II^ in trigonal-pyramidal coordination (Malaestean *et al.*, 2010).

The mononuclear Co(teaH_2_)N_3_ units are linked through O—H···O hydrogen bonding interactions between the ethanol OH groups and the ethanolate O atom of a neighbouring complex into chains running parallel to [010] (Fig. 2).

### Experimental

Under stirring, 2.0 mmol teaH_3_, 4.0 mmol Et_3_N and 4.0 mmol NaN_3_ were added, one after another, into a 20 ml methanol solution containing 1.0 mol Co(ClO_4_)_2_^.^6H_2_O. The resulting solution was kept stirred for another hour, and then filtered. The filtrate was allowed to stand undisturbed in a sealed vessel. Crystallization took place during one week and gave crystals in a yield of 40% based on Co(ClO_4_)_2_^.^6H_2_O. The product was washed with methanol and dried in air.

### Refinement

H1OA and H2OA were found in difference Fourier maps and were refined freely. All other H atoms were positioned geometrically as riding atoms, with C—H = 0.97 Å and with *U*_iso_(H) = 1.2 *U*_eq_(C).

### Crystal data

**Table:**

[Co(C_6_H_14_NO_3_)(N_3_)]	*F*(000) = 516
*M**_r_* = 249.14	*D*_x_ = 1.725 Mg m^−^^3^
Monoclinic, *P*2_1_/*c*	Mo *K*α radiation, λ = 0.71073 Å
Hall symbol: -P 2ybc	Cell parameters from 2179 reflections
*a* = 8.7752 (2) Å	θ = 3.4–27.5°
*b* = 7.9373 (1) Å	µ = 1.78 mm^−^^1^
*c* = 14.4097 (3) Å	*T* = 293 K
β = 107.084 (1)°	Pillar, red
*V* = 959.37 (3) Å^3^	0.20 × 0.20 × 0.10 mm
*Z* = 4	

### Data collection

**Table:**

Rigaku Saturn CCD diffractometer	2179 independent reflections
Radiation source: fine-focus sealed tube	1253 reflections with *I* > 2σ(*I*)
graphite	*R*_int_ = 0.055
Detector resolution: 0.76 pixels mm^-1^	θ_max_ = 27.5°, θ_min_ = 3.5°
ω scans	*h* = −11→11
Absorption correction: multi-scan (*REQAB*; Jacobson, 1998)	*k* = −10→10
*T*_min_ = 0.708, *T*_max_ = 0.823	*l* = −18→18
4004 measured reflections	

### Refinement

**Table:**

Refinement on *F*^2^	Primary atom site location: structure-invariant direct methods
Least-squares matrix: full	Secondary atom site location: difference Fourier map
*R*[*F*^2^ > 2σ(*F*^2^)] = 0.038	Hydrogen site location: inferred from neighbouring sites
*wR*(*F*^2^) = 0.089	H atoms treated by a mixture of independent and constrained refinement
*S* = 0.89	*w* = 1/[σ^2^(*F*_o_^2^) + (0.0427*P*)^2^] where *P* = (*F*_o_^2^ + 2*F*_c_^2^)/3
2179 reflections	(Δ/σ)_max_ = 0.001
135 parameters	Δρ_max_ = 0.55 e Å^−^^3^
0 restraints	Δρ_min_ = −0.38 e Å^−^^3^

### Special details

**Table:**

Geometry. All e.s.d.'s (except the e.s.d. in the dihedral angle between two l.s. planes) are estimated using the full covariance matrix. The cell e.s.d.'s are taken into account individually in the estimation of e.s.d.'s in distances, angles and torsion angles; correlations between e.s.d.'s in cell parameters are only used when they are defined by crystal symmetry. An approximate (isotropic) treatment of cell e.s.d.'s is used for estimating e.s.d.'s involving l.s. planes.
Refinement. Refinement of *F*^2^ against ALL reflections. The weighted *R*-factor *wR* and goodness of fit *S* are based on *F*^2^, conventional *R*-factors *R* are based on *F*, with *F* set to zero for negative *F*^2^. The threshold expression of *F*^2^ > σ(*F*^2^) is used only for calculating *R*-factors(gt) *etc*. and is not relevant to the choice of reflections for refinement. *R*-factors based on *F*^2^ are statistically about twice as large as those based on *F*, and *R*- factors based on ALL data will be even larger.

### Fractional atomic coordinates and isotropic or equivalent isotropic displacement parameters (Å^2^)

**Table:**

	*x*	*y*	*z*	*U*_iso_*/*U*_eq_	
Co1	0.49520 (5)	0.87496 (5)	0.81512 (3)	0.02597 (16)	
C1	0.2117 (4)	0.6897 (4)	0.8302 (3)	0.0438 (10)	
H1A	0.1899	0.6223	0.7716	0.053*	
H1B	0.1173	0.6886	0.8522	0.053*	
C2	0.3480 (4)	0.6158 (5)	0.9063 (3)	0.0405 (9)	
H2A	0.3523	0.6638	0.9690	0.049*	
H2B	0.3329	0.4951	0.9096	0.049*	
C3	0.2225 (5)	0.9829 (4)	0.8818 (3)	0.0424 (10)	
H3A	0.2555	0.9297	0.9451	0.051*	
H3B	0.1099	1.0092	0.8665	0.051*	
C4	0.3151 (4)	1.1428 (4)	0.8848 (3)	0.0362 (9)	
H4A	0.2619	1.2139	0.8301	0.043*	
H4B	0.3217	1.2040	0.9441	0.043*	
C5	0.1604 (4)	0.9162 (5)	0.7086 (3)	0.0423 (10)	
H5A	0.1562	1.0382	0.7049	0.051*	
H5B	0.0518	0.8745	0.6925	0.051*	
C6	0.2386 (4)	0.8496 (5)	0.6363 (2)	0.0361 (9)	
H6A	0.2138	0.7309	0.6249	0.043*	
H6B	0.1970	0.9086	0.5751	0.043*	
N1	0.2476 (3)	0.8648 (3)	0.80846 (19)	0.0254 (6)	
N2	0.7314 (3)	0.8811 (4)	0.8330 (2)	0.0405 (7)	
N3	0.8273 (4)	0.8233 (4)	0.9028 (2)	0.0388 (8)	
N4	0.9226 (5)	0.7663 (5)	0.9675 (3)	0.0703 (12)	
O1	0.4947 (3)	0.6485 (3)	0.88538 (18)	0.0332 (6)	
O2	0.4718 (3)	1.1008 (3)	0.88087 (18)	0.0344 (6)	
O3	0.4087 (2)	0.8714 (3)	0.67098 (14)	0.0269 (5)	
H1OA	0.508 (6)	1.183 (7)	0.863 (4)	0.11 (2)*	
H2OA	0.525 (4)	0.569 (4)	0.870 (3)	0.035 (12)*	

### Atomic displacement parameters (Å^2^)

**Table:**

	*U*^11^	*U*^22^	*U*^33^	*U*^12^	*U*^13^	*U*^23^
Co1	0.0262 (2)	0.0243 (2)	0.0272 (2)	−0.0003 (2)	0.00739 (18)	0.0007 (2)
C1	0.040 (2)	0.0305 (19)	0.064 (3)	−0.0069 (17)	0.021 (2)	0.0004 (18)
C2	0.050 (2)	0.0339 (19)	0.046 (2)	0.002 (2)	0.0274 (19)	0.0055 (19)
C3	0.044 (2)	0.034 (2)	0.059 (2)	−0.0046 (18)	0.030 (2)	−0.0111 (19)
C4	0.042 (2)	0.0266 (19)	0.048 (2)	−0.0025 (18)	0.0257 (18)	−0.0068 (17)
C5	0.029 (2)	0.054 (3)	0.043 (2)	0.0035 (18)	0.0095 (17)	−0.0026 (18)
C6	0.0263 (19)	0.045 (2)	0.0335 (19)	−0.0002 (17)	0.0027 (16)	−0.0022 (17)
N1	0.0282 (14)	0.0220 (13)	0.0280 (14)	−0.0013 (13)	0.0114 (12)	−0.0021 (12)
N2	0.0264 (16)	0.0488 (18)	0.0465 (19)	0.0014 (16)	0.0112 (15)	0.0126 (17)
N3	0.0272 (18)	0.047 (2)	0.045 (2)	−0.0005 (15)	0.0145 (16)	−0.0082 (16)
N4	0.046 (2)	0.107 (3)	0.050 (2)	0.024 (2)	0.001 (2)	0.008 (2)
O1	0.0377 (15)	0.0227 (15)	0.0420 (15)	0.0045 (12)	0.0163 (12)	0.0026 (12)
O2	0.0372 (15)	0.0274 (14)	0.0411 (14)	−0.0091 (12)	0.0152 (11)	−0.0053 (12)
O3	0.0265 (12)	0.0302 (12)	0.0252 (11)	0.0039 (12)	0.0093 (9)	0.0022 (11)

### Geometric parameters (Å, °)

**Table:**

Co1—O3	1.991 (2)	C3—H3B	0.9700
Co1—N2	2.013 (3)	C4—O2	1.433 (4)
Co1—O1	2.064 (2)	C4—H4A	0.9700
Co1—O2	2.065 (2)	C4—H4B	0.9700
Co1—N1	2.148 (3)	C5—N1	1.475 (4)
C1—N1	1.478 (4)	C5—C6	1.502 (5)
C1—C2	1.486 (5)	C5—H5A	0.9700
C1—H1A	0.9700	C5—H5B	0.9700
C1—H1B	0.9700	C6—O3	1.439 (4)
C2—O1	1.430 (4)	C6—H6A	0.9700
C2—H2A	0.9700	C6—H6B	0.9700
C2—H2B	0.9700	N2—N3	1.197 (4)
C3—N1	1.476 (4)	N3—N4	1.147 (4)
C3—C4	1.501 (5)	O1—H2OA	0.74 (3)
C3—H3A	0.9700	O2—H1OA	0.81 (5)
			
O3—Co1—N2	101.32 (11)	O2—C4—H4B	110.0
O3—Co1—O1	116.37 (10)	C3—C4—H4B	110.0
N2—Co1—O1	96.24 (12)	H4A—C4—H4B	108.3
O3—Co1—O2	115.60 (10)	N1—C5—C6	111.6 (3)
N2—Co1—O2	99.08 (12)	N1—C5—H5A	109.3
O1—Co1—O2	121.07 (10)	C6—C5—H5A	109.3
O3—Co1—N1	83.24 (9)	N1—C5—H5B	109.3
N2—Co1—N1	175.37 (11)	C6—C5—H5B	109.3
O1—Co1—N1	80.87 (10)	H5A—C5—H5B	108.0
O2—Co1—N1	79.50 (10)	O3—C6—C5	110.8 (3)
N1—C1—C2	110.6 (3)	O3—C6—H6A	109.5
N1—C1—H1A	109.5	C5—C6—H6A	109.5
C2—C1—H1A	109.5	O3—C6—H6B	109.5
N1—C1—H1B	109.5	C5—C6—H6B	109.5
C2—C1—H1B	109.5	H6A—C6—H6B	108.1
H1A—C1—H1B	108.1	C5—N1—C3	112.2 (3)
O1—C2—C1	110.6 (3)	C5—N1—C1	112.6 (3)
O1—C2—H2A	109.5	C3—N1—C1	111.0 (3)
C1—C2—H2A	109.5	C5—N1—Co1	105.15 (19)
O1—C2—H2B	109.5	C3—N1—Co1	107.8 (2)
C1—C2—H2B	109.5	C1—N1—Co1	107.6 (2)
H2A—C2—H2B	108.1	N3—N2—Co1	122.8 (2)
N1—C3—C4	111.4 (3)	N4—N3—N2	177.5 (4)
N1—C3—H3A	109.4	C2—O1—Co1	113.2 (2)
C4—C3—H3A	109.4	C2—O1—H2OA	109 (3)
N1—C3—H3B	109.4	Co1—O1—H2OA	123 (3)
C4—C3—H3B	109.4	C4—O2—Co1	116.5 (2)
H3A—C3—H3B	108.0	C4—O2—H1OA	107 (4)
O2—C4—C3	108.7 (3)	Co1—O2—H1OA	117 (4)
O2—C4—H4A	110.0	C6—O3—Co1	113.77 (18)
C3—C4—H4A	110.0		

### Hydrogen-bond geometry (Å, °)

**Table:**

*D*—H···*A*	*D*—H	H···*A*	*D*···*A*	*D*—H···*A*
O2—H1OA···O3^i^	0.80 (6)	1.80 (6)	2.595 (3)	176.90
O1—H2OA···O3^ii^	0.74 (3)	1.83 (3)	2.573 (3)	177.70

Symmetry codes: (i) −*x*+1, *y*+1/2, −*z*+3/2; (ii) −*x*+1, *y*−1/2, −*z*+3/2.
